# Ending Lassa neglect: the urgent case for a licensed vaccine

**DOI:** 10.1097/MS9.0000000000005053

**Published:** 2026-04-21

**Authors:** Afnan Ahmad Qureshi, Faiz Ullah, Aleeza Fatima, Muhammad Zaid Saeed, Huzaifa Mahmood, Chisanga Mwape

**Affiliations:** a Department of Internal Medicine, Wah Medical College, Wah Cantt, Pakistan; b Department of Internal Medicine, Kabir Medical College, Peshawar, Pakistan; c Department of Internal Medicine, The Copperbelt University School of Medicine, Ndola, Zambia

**Keywords:** Lassa fever, neglected tropical diseases, outbreak preparedness, ribavirin, vaccine development

## Abstract

Lassa fever is an endemic viral hemorrhagic disease in West Africa with significant morbidity and mortality, yet it remains inadequately addressed in global health priorities. First identified in Nigeria in 1969, the disease continues to affect an estimated 100 000–300 000 individuals annually, with high case fatality rates among hospitalized patients and vulnerable populations. Despite its substantial burden and recurrent seasonal outbreaks, particularly in Nigeria, major gaps persist in both prevention and treatment strategies. Notably, there is no licensed vaccine available more than five decades after its discovery, and current therapeutic practices rely heavily on ribavirin, a drug supported by limited and methodologically weak evidence.

Recent years have witnessed encouraging developments, including early-phase vaccine trials and large-scale epidemiological initiatives such as the ENABLE study, alongside coordinated efforts to establish adaptive clinical trial platforms. However, these advancements remain insufficient without sustained funding, robust clinical trial infrastructure, and policy-level commitment. This letter highlights the urgent need to accelerate vaccine development, reassess existing treatment guidelines, and strengthen outbreak preparedness in endemic regions. Addressing these gaps is critical to transitioning from reactive outbreak response to proactive and evidence-based disease control, ultimately reducing the burden of Lassa fever and improving global health equity.

*Dear Editor*,

This letter adheres to the TITAN 2025 Guidelines for transparent reporting of AI use in scientific communication[1].

Lassa fever is an acute viral hemorrhagic illness first identified in 1969 in northeastern Nigeria, and it remains endemic across several West African countries, including Nigeria, Sierra Leone, Liberia, and Guinea. Caused by an arenavirus and transmitted primarily through exposure to excreta of infected *Mastomys* rodents, the disease also spreads via human-to-human contact in healthcare and community settings. While the majority of infections are asymptomatic, severe cases can progress to multi-organ dysfunction, hemorrhage, and neurological complications. It is estimated that Lassa fever affects between 100 000 and 300 000 individuals annually, with thousands of deaths each year, and case fatality rates reaching 15–20% among hospitalized patients, rising significantly in pregnant women and neonates[2].

Despite this substantial burden, Lassa fever continues to exemplify a neglected tropical disease with critical gaps in prevention and treatment. One of the most striking deficiencies is the absence of a licensed vaccine, even more than five decades after its discovery. This gap is increasingly concerning given the persistence of seasonal outbreaks, particularly in Nigeria, where recent epidemiological data demonstrate both rising case numbers and expanding geographic distribution. Surveillance reports have shown that Lassa fever now affects a majority of Nigerian states, reflecting not only endemicity but also systemic challenges in containment and preparedness[3].

The limitations of treatment further compound the problem. Ribavirin has long been considered the standard of care and is widely administered in endemic regions. However, the evidence supporting its efficacy remains weak and controversial. The recommendation for its use is largely derived from a single study conducted in the 1980s, which has been criticized for methodological limitations, including the lack of proper randomization and reliance on historical controls[4]. More recent systematic reviews have reinforced these concerns, highlighting the high risk of bias across available studies and concluding that there is insufficient high-quality evidence to confirm a definitive survival benefit[5]. This creates a concerning reality in which both primary prevention and definitive treatment are inadequately supported by robust scientific evidence.

Encouragingly, recent years have seen renewed global attention toward Lassa fever research. Several vaccine candidates, including viral vector-based and DNA-based platforms, have entered early-phase clinical development. Early findings suggest promising immunogenicity, however clinical efficacy remains to be established[6]. In parallel, large-scale epidemiological initiatives such as the ENABLE study are improving the understanding of disease burden and transmission dynamics across endemic regions, thereby facilitating more efficient clinical trial planning[7]. In addition, coordinated efforts like the West Africa Lassa Fever Consortium are working to establish adaptive clinical trial platforms capable of evaluating multiple therapeutic candidates in outbreak settings[8].

These developments represent meaningful progress, yet they also underscore the urgent need for the integration of adaptive trial designs into national outbreak response protocols and coordinated action. Accelerating vaccine development must be a global priority, with emphasis on advancing candidates through late-phase trials and ensuring equitable access upon approval. At the same time, there is a pressing need to strengthen clinical trial infrastructure within endemic regions, allowing rapid and ethical evaluation of both existing and novel therapies. Clinical guidelines should be updated to transparently reflect the uncertainty surrounding ribavirin, while actively encouraging patient enrollment into well-designed trials. Finally, public health measures – including improved sanitation, rodent control, and strengthened surveillance systems – remain essential components of outbreak prevention and response[9].

In conclusion, Lassa fever represents a persistent yet addressable challenge in global health. The continued absence of a vaccine, coupled with reliance on inadequately supported therapies, highlights a broader failure to prioritize diseases that disproportionately affect low-resource settings. However, with growing research momentum and increasing international collaboration, there is a clear opportunity to shift from reactive management to proactive, evidence-based control. Bridging this gap will not only reduce the burden of Lassa fever but also serve as a model for addressing other neglected infectious diseases.
